# Correction

**DOI:** 10.1111/cas.14416

**Published:** 2020-05-15

In an article^1^ titled “Bazedoxifene exhibits growth suppressive activity by targeting interleukin‐6/glycoprotein 130/signal transducer and activator of transcription 3 signaling in hepatocellular carcinoma” by Haiyan Ma, Dan Yan, Yina Wang, Wei Shi, Tianshu Liu, Chongqiang Zhao, Shengqi Huo, Jialin Duan, Jingwen Tao, Maocai Zhai, Pengcheng Luo, Junyi Guo, Lei Tian, Lulu Mageta, David Jou, Cuntai Zhang, Chenglong Li, Jiayuh Lin, Jiagao Lv, Sheng Li, and Li Lin, “Tongji Hospital” should be added to affiliation 1 and ‘Departments’ should be changed to its singular form.

Correct authors and their affiliations are listed below

Haiyan Ma^1,2^ | Dan Yan^1^ | Yina Wang^1^ | Wei Shi^1^ | Tianshu Liu^1^ | Chongqiang Zhao^1,3^ | Shengqi Huo^1^ | Jialin Duan^1^ | Jingwen Tao^1^ | Maocai Zhai^1^ | Pengcheng Luo^1^ | Junyi Guo^1^ | Lei Tian^1^ | Lulu Mageta^1^ | David Jou^4^ | Cuntai Zhang^5^ | Chenglong Li^6^ | Jiayuh Lin^7^ | Jiagao Lv^1^ | Sheng Li^1^ | Li Lin^1^

^1^Division of Cardiology, Department of Internal Medicine, Tongji Hospital, Tongji Medical College, Huazhong University of Science and Technology, Wuhan, China

^2^Division of Cardiology, Departments of Internal Medicine, First People's Hospital of ShangQiu, Shangqiu, China

^3^Division of Cardiology, Tianjin First Center Hospital, Tianjin, China

^4^Center for Childhood Cancer, Department of Pediatrics, The Research Institute at Nationwide Children's Hospital, College of Medicine, The Ohio State University, Columbus, OH, USA

^5^Departments of Geriatrics, Tongji Hospital, Tongji Medical College, Huazhong University of Science and Technology, Wuhan, China

^6^Department of Medicinal Chemistry, College of Pharmacy, University of Florida, Gainesville, FL, USA

^7^Department of Biochemistry and Molecular Biology, University of Maryland School of Medicine, Baltimore, MD, USA

The authors apologize for the errors.
